# Consumer perspective on breast density notification in Australia

**DOI:** 10.3332/ecancer.2051

**Published:** 2025-11-28

**Authors:** Sandy Minck, Gerda Evans, Marie Lowe, Cindy Schultz-Ferguson, Catherine Woulfe, Kym Berchtenbreiter, Krysty Sullivan, Ann White, Lynette Moore, Susan Jarvis, Wendy V Ingman, Jennifer Stone

**Affiliations:** 1 Australian Breast Density Consumer Advisory Council (ABDCAC), Perth, WA 6009, Australia; 2 Discipline of Surgical Specialities, Adelaide Medical School, The Queen Elizabeth Hospital, Woodville South, SA 5011, Australia; 3 Robinson Research Institute, University of Adelaide, Adelaide 5005, Australia; 4 Centre for Epidemiology and Biostatistics, Melbourne School of Population Health, University of Melbourne, Melbourne, Victoria 3010, Australia; 5 School of Population and Global Health, University of Western Australia, Perth, Western Australia 6009, Australia; a https://orcid.org/0000-0002-8369-8875; b https://orcid.org/0000-0003-3116-2902; c https://orcid.org/0000-0001-5077-0124

**Keywords:** breast density, risk factors, risk assessment, mammography, health promotion, early detection of cancer

## Abstract

Breast density is a strong and prevalent risk factor for breast cancer and it significantly reduces the sensitivity of mammography to detect the disease. Inconsistency in breast density notification policy across screening programs in Australia has resulted in significant health promotion challenges, with low awareness and knowledge of breast density as a risk factor and confusion regarding assessment and risk management for women with dense breasts. Australian breast density notification policy has recently been revised and now recommends women be informed of their breast density; however, this policy has not yet been implemented in all BreastScreen programs across the country. The research evidence that supports screening policy and practice does not often incorporate the consumer perspective, which is integral to providing clear recommendations for women with dense breasts. Opportunities for consumer organisations to advance evidence-based health promotion strategies to inform public health policy are limited. This article provides a Consumer Perspective on Breast Density Notification from the Australian Breast Density Consumer Advisory Council. This Perspective provides recommendations for future action to support breast density reporting in Australia. It provides a succinct overview, in lay terms, of what breast density is and its role in breast cancer risk assessment, risk-reduction and early detection. Finally, it addresses concerns relating to individual anxiety after receiving a breast density notification. This perspective aims to inform breast screening policy and practice in Australia. It is a position statement on breast density notification from a consumer perspective, built on lived expertise and research evidence. It builds on increasing evidence that inclusion of a consumer voice within medical research and policy development can improve the quality and relevance of health outcomes and provide more effective research translation and impact. This article advances innovation in breast density research and breast screening policy by prioritising the consumer voice, ensuring research and policy are informed by the lived experiences of consumers.

## Introduction

Breast cancer screening saves lives through early detection, leading to improved treatment options [1]. Screening mammography is free for all women in Australia aged 40+ years through the BreastScreen programs, with no referral required. Access to the program includes those recorded as female at birth and gender diverse, in accordance with BreastScreen Australia policy that encourages sensitivity and awareness of gender diversity [2].

Breast density, also known as mammographic density, refers to the white radiological appearance of fibroglandular tissue on a mammogram. Breast density is commonly measured by the Breast Imaging Reporting and Data System (BI-RADS) classification [3] that describes four categories from A to D, where A is almost entirely fatty, B is scattered fibroglandular density, C is heterogeneously dense and D is extremely dense. Approximately 40% of women aged 40–74 have dense breasts (combined categories C and D) and of those, around 10% have extremely dense breasts (category D) [4, 5]; however, distributions may vary by ethnicity [6, 7]. High breast density is associated with an increased risk of breast cancer [8–10] and reduces the ability to detect breast cancer, as both cancers and breast density appear white on a mammogram, making cancers more difficult to see [11, 12].

The Australian Breast Density Consumer Advisory Council (ABDCAC) was established in 2019 to provide a consumer and community perspective on research activities and screening policy to improve breast cancer screening outcomes, particularly activities and policy related to breast density. In Australia, ‘consumer’ or ‘community representative’ are the terms we use to describe people who support healthcare providers, institutions and researchers by providing a patient, caregiver and community perspective. There is increasing evidence and clear value associated with consumer collaborations when informing public health policy design or change [13, 14]. The ABDCAC comprises of 8–12 members with representation from each state, including regional areas of Australia. Members are recruited and vetted via the Western Australian Consumer and Community Involvement Program (https://cciprogram.org/) with oversight from the academic and consumer Co-Chairs and support from a scientific advisor. Members have completed training for effective consumer and community involvement in research. 

Breast density notification is increasingly recognised as a standard of care worldwide. The European Society of Breast Imaging recommends that women be informed of their breast density during screenings [15]. The US Federal Drug Administration mandated that all mammography providers inform patients about their breast density status from September 2024 [16]. Most Canadian provinces now report breast density [17].

Breast density notification policy in Australia has changed considerably since the ABDCAC’s formation. In December 2023, the Royal Australian and New Zealand College of Radiologists updated its Breast Density Position Statement to recommend the reporting of breast density in both screening and diagnostic settings in Australia and New Zealand [18]. BreastScreen Australia’s Breast Density Position Statement from 2020, was that ‘BreastScreen Australia should not routinely record breast density or provide supplemental testing for women with dense breasts. A revised version was released in May 2025, BreastScreen Australia now recommends that ‘Women are informed in writing of their mammographic (breast) density as measured on their screening mammogram.’ An additional recommendation is that ‘BreastScreen Australia clients may seek advice from their general practitioner (GP) or breast cancer specialist regarding whether or how their breast density affects their choice of approach to breast cancer early detection, in the context of their other risk factors, personal circumstances and preferences’ [19]. However, at the time of writing, there are no formal guidelines for Australian GPs when consulting women with dense breasts or the inclusion of breast density information in the Red Book's Guidelines for preventive activities in general practice [20].

Meanwhile, BreastScreen Western Australia has been notifying women if they have dense breasts since 2008. They recommend women with dense breasts consult their doctor to discuss their breast density and have a clinical breast examination [21]. A letter is also sent to their nominated GP, which advises that an ultrasound may be helpful for women with dense breasts who have an additional risk factor, such as a family history of breast cancer. BreastScreen South Australia rolled out statewide breast density reporting to all women in August 2023, using BI-RADS classification categories. They advise women to review their modifiable lifestyle risk factors with their doctor and do not recommend supplemental imaging for asymptomatic women with high breast density who have no other risk factors [22]. BreastScreen Victoria completed its staged roll-out in April 2025 and it recommends that women with extremely dense breasts (BI-RADS Category D) see a doctor for a risk assessment [23]. BreastScreen New South Wales commenced breast density reporting in April 2025. They encourage women who have questions or concerns about their breast density or breast cancer risk to speak with their GP [24]. BreastScreen Australia are working to implement the measurement and reporting of breast density in the remaining Programs, including BreastScreen Australian Capital Territory, BreastScreen Queensland, BreastScreen Northern Territory and BreastScreen Tasmania through a phased approach [25]. Private radiology clinics across the country have been reporting breast density for several years, where supplemental ultrasound is common practice and more recently, contrast enhanced mammography (CEM) is being offered.

The lack of consistent breast density messaging and reporting policies has resulted in confusion regarding risk management for women with dense breasts [26, 27] and potential missed opportunities for early detection of breast cancer for those who are not notified if they have dense breasts. This has also limited opportunities to educate and support women in understanding their breast health and reducing their risk of breast cancer. The ABDCAC believes that a consumer perspective should be integral to a national approach to the implementation of breast density notification in Australia. This article provides a succinct Consumer Perspective on breast density notification, co-developed by the ABDCAC in consultation with leading academic experts, to inform breast density screening policy in Australia.

## Methods

This Consumer Perspective on Breast Density Notification was developed by the ABDCAC in collaboration with two leading experts in breast density research (JS and WI). A working group within the ABDCAC was established and a framework for the Perspective was drafted by an ABDCAC member (SM) and circulated to the working group for review. After several iterations, the draft was circulated to all ABDCAC members for further review. The final draft of the Perspective was reviewed by the ABDCAC academic Co-Chair (JS) and the supporting manuscript was drafted. The manuscript was finalised through the same iterative process.

**Consumer perspective on breast density notification**

**About us**

The ABDCAC was established to:

- provide consumer and community perspectives on the research activities across Australian and international institutions interested in breast density research and breast cancer screening,
- provide consumer and community expertise and advice on issues and priorities for research, including advocacy where appropriate, and
- communicate research findings to the wider community.

The purpose of this document is to inform breast screening policy in Australia by providing a consumer perspective on breast density notification, emphasising its importance in breast cancer risk assessment, risk-reduction strategies and early detection.

**Recommendations**

The ABDCAC calls for the following actions from the Australian Commonwealth Government:

1. A national approach to breast density notification: Report breast density in all screening and diagnostic mammogram reports in a clear and consistent way across Australia. This should, for now, include the risks associated with breast density, the individual's BI-RADS category of density and potentially in future, a mammogram-based risk score.
2. Develop health promotion resources: Partner with consumer and community groups to develop easy-to-understand and culturally appropriate information and educational materials in multiple formats.
3. Increase awareness of breast density: Promote information about breast cancer risk factors to the Australian community so that women have more opportunity to learn about breast density before they are notified of their own breast density category.
4. Develop pathways for health professionals: Co-develop pathways for supplemental imaging options for women with dense breasts and those at above average risk of breast cancer.
5. Improve availability and access to medical imaging: Increase government funding to improve equitable access to breast imaging tools such as tomosynthesis (3D mammography), ultrasound, CEM and magnetic resonance imaging (MRI).
6. Enhance breast density research: Fund breast density research, including risk-assessment, risk-reducing strategies and early detection of breast cancer. Those with lived experience should be partners in all stages of the research process.
7. Align with cancer health policy: Ensure Australia-wide screening practices align with the Australian Cancer Plan, the Aboriginal and Torres Strait Islander Cancer Plan, the Optimal Care Pathway for Breast Cancer and other national health strategies.

**Rationale for recommendations**

Notifying women of their breast density will enable:

- increased knowledge and awareness of associated risk of breast cancer and risk of cancers going undetected at mammographic screening (masking),
- women to participate in decision-making about how to best manage their breast health,
- individual breast cancer risk assessment using validated tools like iPrevent,™[50]
- discussion with health care professionals about risk-reducing strategies, such as lifestyle changes or medication for women at moderate or high risk,
- early detection of breast cancer via discussions with health care professionals about whether supplemental screening is recommended (e.g., tomosynthesis, ultrasound, CEM and MRI).

**What everyone should know about breast density**

Breasts are made up of fatty tissue and fibroglandular tissue (glands that make milk, held together by fibrous tissue). A mammogram is an X-ray of the breast, with fibroglandular tissue appearing white and fatty tissue appearing dark. The white areas are referred to as breast density.

Breast density is commonly measured using the BI-RADS® (5th edition). There are four categories:

- Category A: The breasts are almost entirely fatty.
- Category B: There are scattered areas of fibroglandular density.
- Category C: The breasts are heterogeneously dense, which may obscure small masses.
- Category D: The breasts are extremely dense, which lowers the sensitivity of mammography.

Dense breast tissue is common, with approximately 40% of Australian women aged 40–74 in category C or D, with ~10% having extremely dense breasts (category D) [5].

Breast density decreases with age, but a woman’s risk of breast cancer increases with age. This is why it is important to consider a woman’s age, and her other risk factors, when discussing recommendations regarding breast health [18].

Breast density cannot be determined by feel or touch.

**Associated risks of 1.) Breast cancer and 2.) Masking**

Women with extremely dense breasts (Category D) are:

- twice as likely to develop breast cancer compared to those of the same age with scattered density (Category B) [8];
- and four to six times more likely to develop breast cancer than those of the same age with fatty breasts (Category A) [9, 10].

Both breast density and cancers appear white on a mammogram, making cancers harder to see (known as the masking effect). This could lead to a false negative screening result (getting an all clear when cancer is present). The ‘sensitivity’ of screening mammography to detect breast cancer is the percentage of women who attend screening and have breast cancer who are correctly identified as having breast cancer. The sensitivity of mammography for women with extremely dense breasts (Category D) is around 63%, compared to around 93% for women with fatty breasts (Category A) [12, 28].

**Risk assessment**

There are risk assessment tools that use breast density to calculate the individual risk of breast cancer. The iPrevent tool (www.iprevent.net.au) is endorsed by Cancer Australia. The online assessment can be completed by women or in consultation with a health professional. There are currently other mammography measures being developed using artificial intelligence that may also help identify women at increased risk of breast cancer in the future, using a mammogram-based risk score [29].

**Risk-reducing strategies**

The most substantial evidence for reducing breast density and breast cancer risk involves taking daily medication [30]. This is only recommended for women at moderate or high risk of breast cancer [31].

**Early detection**

Additional imaging tests or ‘supplemental screening’ may benefit women with dense breasts, particularly if they have another risk factor for breast cancer. In Australia, supplemental screening includes tomosynthesis, ultrasound, CEM and MRI. Other breast cancer risk factors include age, family history, genetic markers, personal history of breast or ovarian cancer, previous biopsies, mantle radiation for lymphoma, certain hormone therapies and lifestyle factors such as body mass index and alcohol consumption [32].

There is currently no consensus in Australia on post-screening recommendations for women with dense breasts or guidelines for health professionals when consulting women who have received a breast density notification.

**Addressing concerns about anxiety**

Anxiety and psychosocial harms associated with breast density notification continue to be areas of research [26, 33]. The ABDCAC contends that concerns about breast density notification causing anxiety are not supported by most women and that provision of information about breast density should not be considered a harm. Research indicates that the anxiety associated with high breast density is often situational and can act as a motivator, encouraging women to be more vigilant with routine mammographic screening and reducing their risk of breast cancer [27, 34].

**Conclusion**

Breast density notification is essential for empowering women to optimally manage their breast health. By providing clear, evidence-based information and enabling informed decision-making, we can improve breast cancer risk assessment, risk reduction strategies and early detection of breast cancer for all women in Australia.

## Discussion

Research consistently reports that the majority of women want to be informed of their breast density [4, 26, 35, 36]. Breast density notification enables women to make informed decisions on how to best manage their breast cancer risk and screening [37]. This Consumer Perspective on Breast Density Notification provides a contemporary position statement, written in language that is easy to understand and outlines seven recommendations to support a national approach to the implementation of breast density notification in Australia. Recommendations include consumer-friendly health promotion resources for breast density reporting and clear guidance for health professionals and women with dense breasts. It can be used by women, researchers, health professionals, industry and screening policy makers to improve breast density education and inform future policy decisions.

Breast density notification is consistent with best practices in informed decision-making as stated by the Breast Screen Australia Accreditation Review Committee: ‘Best practice ensures that women who participate in the Breast Screen Australia Program are fully informed about breast cancer screening, including the likely benefits and possible harms, as well as any risks or uncertainties related to the screening process. The information provided will need to be sufficient to enable women to give their informed consent to participate in screening and to undergo any assessment investigations that may be required’ [38]. The ABDCAC contends that breast density notification enables women to be best equipped with the information needed to make informed decisions about breast cancer screening and their breast health.

Breast density notification is also an opportunity to increase awareness of breast health and breast cancer risk factors within asymptomatic women. Current evidence indicates that women’s knowledge and awareness of breast density as a breast cancer risk factor is low [4, 26, 35]. This is consistent with a literature review commissioned by Breast Screen Australia that reported there was limited research about Australian women’s knowledge of general and specific breast cancer risk factors [39]. Breast density is one of many risk factors for breast cancer. Breast density notification could start conversations about individual breast cancer risk assessment and personalised breast health management strategies.

There is increasing evidence that suggests more tailored approaches to screening could lead to more efficient and effective screening [40–42]. The current ‘one-size-fits all’ approach leaves considerable room for improvement in screening participation, evidenced by the number of women who are unnecessarily recalled for further assessment and the number of interval cancers that are diagnosed within the 12–24 months after getting the all-clear [43]. There are new mammographic measures that are currently being developed using artificial intelligence that may not only help detect cancer at screening but also help identify those at increased risk of future breast cancer [44–46]. Improved knowledge and discussion regarding breast density-associated risks could also pave the way for an understanding of future mammogram risk scores that could revolutionise breast screening.

It is anticipated that more efficient and effective screening could lead to increased participation in breast screening, with national screening participation currently around 50% for women in the targeted age range of 50–74 years [43]. An Australian study has shown that breast density notification does not deter women in the targeted age range from attending subsequent screening, reporting that women screening for the first time who were notified of their breast density were more likely to return to screening when next due than those not notified [47].

Knowledge of risk factors may also empower women to estimate their individual risk and explore risk-reducing strategies. For example, iPrevent™ [48] is an online decision support tool for breast cancer risk assessment and risk management that may help facilitate prevention and screening discussions between women and their healthcare professionals [48]. It uses women’s risk factor information, including their breast density, to calculate their risk of developing breast cancer in their lifetime or in the next 10 years. It does not replace a medical consultation. It can take up to 30 minutes to answer all the questions. A summary document is produced at the end, which shows an individual’s risk compared to the general population and provides risk management information. This could be printed and/or emailed to the healthcare professional prior to the consultation. Risk-reducing strategies may include maintaining a healthy body weight, reducing alcohol consumption and in some cases considering medication options.

Information and resources for health professionals, particularly GPs, currently differ by the source of information and/or the state in which they practice. The information also lacks clear guidance regarding what to recommend for women with dense breasts. This is partly because recommendations that involve supplemental imaging raise concerns with health equity, access and cost. However, doing nothing could make things worse. A national approach to breast density reporting is needed to ensure consistent messaging for women with dense breasts and their consulting health professionals. BreastScreen Australia is currently undergoing a National Policy and Funding Review [49]. One of its goals is to enable a nationally consistent approach to breast screening practices. It is hoped that one of the review’s priorities will include breast density notification, including funding to support the implementation of automated breast density measurement software and improved availability and access to medical imaging. Prevention and early detection of breast cancer is a key component of the Australian Cancer Plan, the Aboriginal and Torres Strait Islander Cancer Plan and the Optimal Care Pathway for Breast Cancer. Breast density notification is a vital component of prevention and early detection and can improve knowledge and awareness about breast cancer risk. It can help assess individual risk and increase uptake of screening and risk-reducing strategies.

## Conclusion

The ABDCAC is committed to advocating for policies and practices that prioritise consumer and community needs and enhance public health. The ABDCAC supports the premise that all women have a right to information that may inform their individual breast cancer risk, including breast density. The ABDCAC recommends breast density notification in all screening and diagnostic mammography settings to support personalised risk assessment and empower women with information to make informed decisions about their breast health. We advocate for consumer-driven design of communication, education and support strategies to ensure the information provided meets the needs of those for whom it is intended. By providing clear, evidence-based information, and enabling informed decision-making, we can improve breast cancer risk assessment, risk reduction strategies and early detection of breast cancer for all women in Australia.

## List of abbreviations

ABDCAC, Australian Breast Density Consumer Advisory Council; BI-RADS, Breast imaging reporting and data system; CEM, Contrast enhanced mammography; GP, General practitioner; MRI, Magnetic resonance imaging.

## Conflicts of interest

No conflicts to declare.
